# Recent Insights on Biological and Ecological Aspects of Ectomycorrhizal Fungi and Their Interactions

**DOI:** 10.3389/fmicb.2018.00216

**Published:** 2018-02-15

**Authors:** Antonietta Mello, Raffaella Balestrini

**Affiliations:** Institute for Sustainable Plant Protection (IPSP), Torino Unit, National Research Council, Turin, Italy

**Keywords:** ectomycorrhizae, plant–microbe interactions, symbiosis, cell wall, mycorrhizal fungi

## Abstract

The roots of most terrestrial plants are colonized by mycorrhizal fungi. They play a key role in terrestrial environments influencing soil structure and ecosystem functionality. Around them a peculiar region, the mycorrhizosphere, develops. This is a very dynamic environment where plants, soil and microorganisms interact. Interest in this fascinating environment has increased over the years. For a long period the knowledge of the microbial populations in the rhizosphere has been limited, because they have always been studied by traditional culture-based techniques. These methods, which only allow the study of cultured microorganisms, do not allow the characterization of most organisms existing in nature. The introduction in the last few years of methodologies that are independent of culture techniques has bypassed this limitation. This together with the development of high-throughput molecular tools has given new insights into the biology, evolution, and biodiversity of mycorrhizal associations, as well as, the molecular dialog between plants and fungi. The genomes of many mycorrhizal fungal species have been sequenced so far allowing to better understanding the lifestyle of these fungi, their sexual reproduction modalities and metabolic functions. The possibility to detect the mycelium and the mycorrhizae of heterothallic fungi has also allowed to follow the spatial and temporal distributional patterns of strains of different mating types. On the other hand, the availability of the genome sequencing from several mycorrhizal fungi with a different lifestyle, or belonging to different groups, allowed to verify the common feature of the mycorrhizal symbiosis as well as the differences on how different mycorrhizal species interact and dialog with the plant. Here, we will consider the aspects described before, mainly focusing on ectomycorrhizal fungi and their interactions with plants and other soil microorganisms.

## Introduction

The roots of most terrestrial plants are colonized by mycorrhizal fungi. They play a key role in terrestrial environments providing to plants an improvement in mineral nutrient uptake and earning in return carbon compounds (Brundrett, 2009). Mycorrhizal interactions are usually classified on the basis of the features of the symbiotic interfaces and of the taxonomic identity of the plant and fungal symbionts (Smith and Read, 2008). Among mycorrhizal symbioses (see van der Heijden et al., 2015 for a review), ectomycorrhizae are established by the mycelia of fungi almost exclusively belonging to the so called “higher fungi," i.e., Basidiomycetes and Ascomycetes, whose ecological strategies have been revisited by Tedersoo and Smith (2013). Ectomycorrhizal (ECM) fungi are present all over the world, and their host plants include most angiosperm and gymnosperm trees, as well as shrubs (Bonfante, 2010). Some ECM plants are economically important timber-producing tree species, while some ECM fungi are represented by the economically important truffles and porcini (Mello et al., 2015a). Between plant and soil there is a very specific environment, the ectomycorrhizosphere, in which diverse communities of microorganisms – fungi and bacteria – interact. It is known that ECM fungi have a key role in nitrogen cycling, particularly in boreal and temperate forests, and that they can help their host plants to tolerate abiotic stresses. ECM assemblages provide benefits for inorganic nitrogen uptake under environmental constraints, through stress activation of distinct ECM fungal taxa. This suggests that these taxa are functionally diverse and opens new opportunities to characterize the ECM fungal identities (Pena and Polle, 2014). Furthermore, Gehring et al. (2017) demonstrated that tree genetics determines fungal partner communities that confer drought tolerance, highlighting the interlinked importance of the genetics of a tree and its microbiome.

The development of an ECM symbiosis requires morphological changes in the two partners, to allow the formation of the symbiotic structures, through the regulation of several genes (Martin et al., 2007; Kohler et al., 2015). From the first work in which cDNA arrays were used to study gene expression in the ECM symbiosis between *Eucalyptus globulus* and *Pisolithus tinctorius* (Voiblet et al., 2001), important progress has been done in the comprehension of the mechanisms involved in the ectomycorrhiza development. Information on the functional diversity of the ECM interactions has been highlighted, leading to the discovery of many genes coding for plant/fungus symbiosis-regulated proteins. Among them, several mycorrhiza-induced small-secreted proteins (MiSSPs) that may act as effectors and are required for symbiosis establishment have been identified (Plett et al., 2011, 2014a; Kohler et al., 2015; Martin et al., 2016 for a review). Additionally, Pellegrin et al. (2015) showed, through a bioinformatics pipeline, that the secretome of ECM fungi is enriched in SSPs in comparison to other species with a different life style. Shared- and lifestyle-specific SSPs have been identified in saprotrophic and ECM fungi, and the ECM-specific SSPs could be a signature of the ECM symbiosis lifestyle. This would suggest they have a role in a molecular dialog with host plants, leading to the formation of a functional ectomycorrhiza (Pellegrin et al., 2015; Garcia and Ané, 2016 for a commentary).

Despite similar anatomical patterns, the sequenced ECM genomes showed that differences are present in symbiosis regulated genes, revealing a diversity in the manner by which symbiotic fungi interact with their partners and suggesting the use of different molecular toolboxes to dialog with the host plant (Martin et al., 2008, 2010; Kohler et al., 2015; Peter et al., 2016). Remarkably, the role of MiSSPs (such as MiSSP7) to control host plant defense reactions has been elegantly demonstrated in *Laccaria bicolor* and *Populus trichocarpa* interaction (Plett et al., 2011, 2014b), while such fungal effectors have not been found among the upregulated transcripts in *Tuber melanosporum* ECMs (Martin et al., 2010), suggesting that different mechanisms may be involved in the development and maintaining of the ECM symbiosis. Transcript profiling of ECM roots from different plant/fungus interactions suggests that similar functional gene categories appear to be up-regulated, although these genes are not the same in the several ECM fungal species (Kohler et al., 2015). The availability of more genome sequences from ECM fungi also confirms that they have a reduced set of genes encoding plant cell wall degrading enzymes (PCWDEs) (Kohler et al., 2015), as already suggested from the genome sequence of the first two sequenced mycorrhizal fungi, i.e., *L. bicolor* and the black truffle *T. melanosporum*, respectively (Martin et al., 2008, 2010). In addition to genomic features and transcriptomic profiles, epigenetic variation is considered an important player in the evolution of biological diversity, and epigenetic regulatory systems have an important role in the response to environmental stimuli and stress factors (Zhong, 2016). The availability of the genome sequences from several fungal species will allow the understanding on how DNA methylation regulatory components are evolved in ECM fungi, and the role of the epigenetic mechanisms to cope with different environmental conditions through modifications of gene expression mediated by DNA methylation and transposon activity profiles. Considering that DNA methylation in fungi lead to transposable elements (TEs) silencing, comparative methylome and transcriptome analyses have been performed in a TE-rich organism such as the ECM fungus *T. melanosporum* (Chen et al., 2014), suggesting that a reversible methylation mechanism functions in truffles to cope with the multitude of TEs present in its genome. Information derived from these analyses, whether extended to individuals from different geographical areas, may also provide a new tool to explain intraspecific variability and adaptation to different environments and, in the case of truffles, commercially organoleptic properties (i.e., aroma).

An ECM root is a complex organ, formed not only by two individuals, plant and fungus, but also by two fungal pseudotissues: the mantle (i.e., the sheat), which develops outside the root, and the Hartig net, which colonizes the apoplastic space between root cells (Balestrini et al., 2012; Balestrini and Kottke, 2016). The two ECM compartments are thought to be functionally different. This has been first demonstrated by a study on *Amanita muscaria* ectomycorrhizae, where the mantle was manually dissected from the ectomycorrhizal root, revealing a differential expression for two fungal genes coding for a phenylalanine ammonium lyase (*AmPAL*) and a hexose transporter (*AmMst1*) (Nehls et al., 2001). While the first (*AmPAL*) was mainly expressed in the mantle, the expression of *AmMst1* increased in the Hartig net. More recently, the combination of a laser microdissection (LMD) approach, which allows the collection of the two ECM fungal compartments, with microarray gene expression analysis, revealed a specificity in the transcript profiles, reflecting a functional specificity for these two ECM compartments, e.g., that the mantle is the responsible for the mineral elements (i.e., nitrogen) and water uptake from soil, while the expression of several transporters is enhanced in the Hartig net (Hacquard et al., 2013). In the last years, different reviews have been focused on the molecular signals (mechanisms) underlying the ECM development and functioning (Garcia et al., 2015; Martin et al., 2016). A role of flavonoids and hormones in the signaling pathway during the early stages of the ECM establishment has been proposed since several years (Garcia et al., 2015 and references therein). More recently, two plant flavonoids have been suggested to trigger the expression of a fungal effector (MiSSP7, see below) in *L. bicolor* (Plett et al., 2014a). It has been also reported that accumulation at the root apex and redistribution of auxin, which is a hormone produced by both the symbiotic partners, may play a role to stimulate lateral short root development required for the ECM formation (Felten et al., 2009). Martin et al. (2016) have recently speculated that secreted fungal MiSSPs may interact with auxin, gibberellin, and salicylate receptors to alter root development. Moreover, increased concentrations of ethylene and jasmonic acid repressed fungal colonization, with an impact on the development of the ECM roots (Plett et al., 2014b). However, the effective involvement of plant hormones in ECM establishment and maintenance has to be still fully elucidated (Garcia et al., 2015).

Here, some specific aspects related to the biology and ecology of the ECM fungi will be considered, starting with their *in situ* dynamics to the symbiotic interface creation, before and after their genome sequencing and the advent of the environmental genomics.

Given that truffles are of high economic interest, crossing several research fields ranging from taxonomy to truffle cultivation, and are the first edible ECM fungi whose genome has been sequenced, extensive research has been focused on them in order to understand their life cycle and thus to increase their production. For this reason, particular attention will be given to some insights highlighted by the sequencing of the black truffle *T. melanosporum* genome.

## Identification of Ectomycorrhizal Fungi: From the Past to the Present

The identification of ECM fungi has generally been focused on the macro- and microscopic examination of fruiting bodies and only since the early 1990s these fungi have also been characterized by DNA-based methods. At the beginning, most of the identification of fruiting bodies has involved restriction analyses of the internal transcribed spacer (ITS) region producing ITS-RFLP database from sporocarp samples (Horton and Bruns, 2001). The next step has been the direct sequence analysis of the ITS region and its deposit in GenBank or EMBL. Specific primers have been then developed for the identification of many fungal species upon increase of sequences number (Gardes et al., 1991). Amicucci et al. (1998) designed ITS primers for the identification of five species of white truffles, *T. magnatum* Pico, *T. borchii* Vittad., *T. maculatum* Vittad., *T. dryophilum* Berk. & Br. and *T. puberulum* Berk. & Br. that have similar morphological characteristics, but different organoleptic qualities and economic value. At this regard, Mello et al. (2006) designed ITS primers for the identification of the marketable boletes *Boletus edulis* Bull.: Fr. *sensu stricto*, *B. aereus* Bull.: Fr, *B. pinophilus* Pila ìt et Dermek and *B. aestivalis* Fr. (all classified as *B. edulis* s.l.), which are hardly distinguishable on the basis of their morphology and considered as the most frequently eaten fungi among those harvested in natural conditions in Europe. Once the molecular tools as sequencing and specific primers have been available, they have allowed typing the ECM tips, usually after sorting these in morphotypes. This method has, thereafter, been used in many studies on EM community structure and spatial distribution since the pioneering work of Gardes et al. (1991). According to Kaldorf et al. (2004), roughly 90% of all ectomycorrhizas of aspen clones in experimental fields was represented by *Cenococcum geophilum*, *Laccaria* sp., *Phialocephala fortinii*, two different Thelephoraceae, and one member of the Pezizales. Murat et al. (2005) sorted 335 mycorrhizal root tips collected in a truffle-ground into 39 morphotypes, on the basis of color, mantle shape and surface texture, presence of cystidia, and EM branching pattern, providing novel information on the ectomycorrhizal and endophytic species living in a *T. magnatum* truffle-ground. Above all, the finding of the few *T. magnatum* mycorrhizae in a non-productive period for this fungus, and in a non-productive area, suggested that there is not a direct linkage between mycorrhizae and fruiting bodies. Since mycorrhizal networks permit interactions among trees, their architecture has been investigated by multi-locus microsatellite DNA, leading to the identification of the trees and fungal genets connected in a multi-aged old-growth forest (Beiler et al., 2010). In order to study functional diversity among ECM fungi *in situ*, the activities of enzymes involved in the degradation and nutrient release from soil organic matter have been used (Courty et al., 2010; Pritsch and Garbaye, 2011). Combining enzymatic activities and stable isotope assays of root tips Tedersoo et al. (2012) have tried to assess the functional aspects of tropical ECM fungi. This study demonstrates that the ECM fungus may affect both potential enzymatic activities and δ^15^N patterns of ECM tips in relation to phylogeny and exploration type (i.e., contact, short distance, medium-distance fringe and long-distance types; cf. Agerer, 2001).

As each ECM species is specialized in exploiting specific resources of the soil ecosystem, investigations have been thereafter focused on the spatial distribution of the extraradical mycelium. It interconnects plant rootlets in the forest ecosystems, forming the ‘wood wide web’ (Martin et al., 2016). Tracking the distribution of a given ECM fungus is considered difficult, since fruiting bodies do not reflect the distribution of ground networks (Dahlberg, 2001). The detection of the mycelium in soil has been possible thanks to the advent of new methods that have led to the direct extraction and amplification of DNA from this environment. *Hebeloma cylindrosporum* was the first ECM fungus to be detected in soil, within 50 cm from the fruiting bodies (Guidot et al., 2002). Using the β-tubulin gene as a marker, Zampieri et al. (2010) could show that the mycelium of *T. magnatum* is more widespread than was inferred from the distribution of its fruiting bodies and ectomycorrhizae. Thanks to the progress of real-time PCR techniques, that has been optimized to quantify ECM mycelium of several ECM fungi (Iotti et al., 2014), De la Varga et al. (2012) quantified *B. edulis* extraradical mycelium in a Scots pine forest and found positive correlation between the concentration of mycelia and the presence of mycorrhizae of *B. edulis*, but not with the productivity of fruiting bodies, in the investigated samples. Given that knowledge of the annual dynamics of the mycelium of ectomycorrhizal fungi in forests soils is important in the carbon cycle, the mobilization of soil nutrients, and in the interactions of different components (plants, fungi, microfauna, and microorganisms) of the ecosystem, De la Varga et al. (2013) investigated with the same approach, the seasonal dynamics of *B. edulis* and *Lactarius deliciosus* extraradical mycelium in pine forests of central Spain. Soil mycelial dynamics of both species resulted to be strongly dependent on the weather, with an increase of biomass during the coldest months of the year.

Once it has been possible detecting mycelium of a given species and tracing its distribution, research has moved toward genet localization not only of sporocarps but also of the subterranean parts, i.e., the ectomycorrhizae and the extraradical mycelia. Studies based on the analysis of the genet structure of sporocarps, have proved that early stage fungi such as *Hebeloma* and *Laccaria* formed many small genets, and late stage fungi such as *Cortinarius* formed a few large genets (Hirose et al., 2004). Using a polymorphic microsatellite marker specific for *Suillus grevillei*, Zhou et al. (2001) demonstrated that the development of *S. grevillei* sporocarps is correlated with that of extra-radical mycelia and ectomycorrhizae of the same genet, which are distributed in a narrow area, however, no *S. grevillei* mycelia and mycorrhizae were detected close to the area where *S. grevillei* sporocarps emerged in the previous year, thus suggesting that subterranean genets change location year after year. Also Guidot et al. (2001) found a spatial congruence of above- and below-ground distribution for *H. cylindrosporum*, and the tendency of the same genets to be dominant above and below ground. Interestingly, it has been possible proving the interconnection between a single genet and different plants. At this regard, Lian et al. (2006) showed that each genet detected in the mycelial mats of *Tricholoma matsutake* colonized from three to seven trees in a natural *Pinus densiflora* forest.

All these studies have been focused on the individual recognition of ectomycorrhizal fungi clarifying many aspects of their population biology (for a review see Dowhan et al., 2011). The introduction of high-throughput sequencing techniques and the suitability of studying (micro)organisms directly *in situ* (metagenomics or environmental genomics) has provided new information on ECM fungal communities by ‘barcodes’ of ITS regions in several biomes/ecosystems, e.g., tropical African forests (Tedersoo et al., 2010); Swedish spruce plantations (Wallander et al., 2010); truffle grounds (Mello et al., 2011); transgenic poplar plantations (Danielsen et al., 2012); ECM roots in the Svalbard (Blaalid et al., 2012, 2014); an urban landscape (Lothamer et al., 2014); boreal and tropical forests (Clemmensen et al., 2013; McGuire et al., 2013a); a forest dominated by oaks in Japan (Toju et al., 2013); *Pinus sylvestris*-dominated plots across three study areas in Estonia (Hiiesalu et al., 2017).

In parallel with the development of the next-generation sequencing systems such as 454 Genome sequencer (introduced in 2005, it uses real-time sequencing-by-synthesis pyrosequencing technology), the Illumina platform (utilizes a sequencing-by-synthesis approach coupled with bridge amplification on the surface of a flow cell) and Ion Torrent PGM (relies on the real-time detection of hydrogen ion concentration, released as a by-product when a nucleotide is incorporated into a strand of DNA by the polymerase action), new tools such as FUNGuild, have been developed to taxonomically parse fungal OTUs by ecological guild independent of sequencing platform or analysis pipeline. Using a database and an accompanying bioinformatics script, Nguyen et al. (2015) demonstrated the application of FUNGuild to three high-throughput sequencing datasets from different habitats: forest soils, grassland soils, and decomposing wood. Several pipelines provided as web services have been produced for processing fungal ITS metabarcoding using 454-sequenced amplicons, such as CLOTU (Kumar et al., 2011), SCATA^1^, PLUTOF (Abarenkov et al., 2010). Once the Illumina MiSeq platform for fungal metabarcoding has become very popular (starting with research by Bokulich et al., 2013; McGuire et al., 2013b; Schmidt et al., 2013), Bálint et al. (2014) developed a pipeline for cleaning up fungal ITS metabarcoding data generated on this platform.

An investigation of community–environment relationships in truffle grounds of the ECM fungus *T. melanosporum*, sampled in two areas, one devoid of vegetation (known as brulé in French and where fruitingbodies of this fungus are usually collected), and outside the brulé, has shown that Ascomycota were the dominant phylum in the brulé, and that their number decreased moving from inside the brulé to outside, while the number of Basidiomycota increased (Mello et al., 2011). Furthermore, this work provides comparison of the two ITS regions, ITS1 and ITS2, for fungal communities assessment. Changes in ECM fungal communities have been registered in many investigations (Mello et al., 2015b). Hui et al. (2011) have observed, in a Finnish forest, that a long-term exposition to Pb contamination can result in a shift in the composition of the ECM community associated with *P. sylvestris* L., as well as an increase in the abundance of the OTUs corresponding to the *Thelephora* genus and a decrease in the frequency of OTUs assigned to *Pseudotomentella*, *Suillus*, and *Tylospora*. In the Siberian tundra Gittel et al. (2013) have verified a decrease in ECM fungi abundance and an increase of bacteria in buried soils because of the low temperature and anoxia of these sites. ECM fungal communities of a temperate oak forest soil resulted to be affected by seasonality and soil depth (Vořìšková et al., 2014). Rincón et al. (2015) revealed a highly compartmentalized and contrasted response of fungal communities of *Pinus sylvestris* in France and Spain with different response of fungal sub-assemblages in soil vs. roots and lifestyle. High-throughput sequencing analysis of fungal communities in temperate beech forests in Germany showed that distance decays of soil-inhabiting and root-associated fungal assemblages differ, and identified explanatory environmental variables (Goldmann et al., 2016).

**FIGURE 1:**
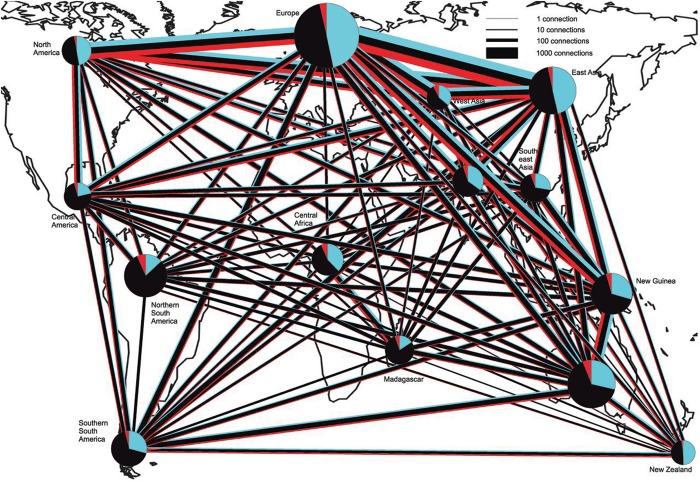
Connectedness of biogeographic regions by shared OTUs of EcM fungi, saprotrophs, and plant pathogens. Blue, EcM fungi; Black, saprotrophs; and red, plant pathogens. The width of lines and diameter of pie charts are proportional to the square root of the number of connections and sample size (number of sites), respectively. Pie charts indicate the number of OTUs found in each region. OTUs with a single sequence per site and OTUs belonging to Hypocreales and Trichocomaceae (in which the ITS region is too conservative for species-level discrimination) were excluded. Figure modified from Tedersoo et al. (2014).

Although many sophisticated bioinformatics tools are available, high-throughput assessment of species richness and evenness in a fungal community (including ectomycorrhizal fungi) remains still a technical challenge, because of the methodological biases and the limitations of markers. According to Lindahl et al. (2013) the major benefit of high-throughput methods relies in their capacity to unearth the main fungal colonizers in large numbers of samples, since not always singletons represent authentic rare taxa. A global soil sampling in 365 sites across the world, followed by DNA metabarcoding, revealed representatives of all major phyla and classes of fungi in all ecosystems but with relative proportions variable several fold across biomes, in addition to several deeply divergent class-level fungal lineages that had not yet been described or previously sequenced (Tedersoo et al., 2014). The overall richness of soil fungi increased toward the equator, however, functional differences were observed between fungal communities in forested and tree-less ecosystems. In fact, richness of ECM fungi head a peak at mid latitudes, especially in temperate forests and Mediterranean biomes of the Northern Hemisphere, in accordance with the dominance at mid latitudes of Pinaceae, which is the oldest family of ECM plants (**Figure 1**). Data from this paper clearly determine climatic factors as the main drivers of fungal diversity and community composition, and greatly advance our understanding of global fungal diversity patterns. Beside this, they alert us on the impact of climate change on the consequences of altered soil microorganism communities and highlight the lack of data from understudied tropical and subtropical ecosystems (Tedersoo et al., 2014). Moving from a global scale analysis to the tripartite associations between roots, fungi and bacteria, that are known to influence plant health and growth (Bonfante and Anca, 2009), Marupakula et al. (2017) identify the different bacterial communities associated with different types of ECM associations, providing clue for more detailed functional studies of specific combinations of ECM fungi and bacteria. Furthermore the study shows as N additions impact fungal–bacterial interactions at the ectomycorrhizal root tip level in different soil horizons, likely influencing patterns of carbon allocation to roots. The study of microbe–microbe interactions is recently also taking advantage of a combination of -omics with direct process measurements (e.g., stable isotope probing ‘SIP’) to map functions and relationships in complex communities. Musat et al. (2016) review how is might be possible now to track microbial interactions with NanoSIMS (Nano secondary ion mass spectrometry), that has the potential to provide quantitative measures of organic matter-mineral-microbial interactions and biogeochemical processing at the macro- and microaggregate or single-cell scale. Regarding this approach, Worrich et al. (2017) demonstrate that mycelium-forming fungi and oomycetes provide nitrogen, carbon and water to bacteria in dry and oligotrophic environments, thus helping them and contributing to ecosystem functioning in stressed conditions.

Anyway, the parallel increase of data output from high-throughput sequencing and of databases of entire genomes will move the research toward direct analysis of meta-genomes and meta-transcriptomes of complex fungal communities (Kuske and Lindahl, 2013). However, Marmeisse et al. (2013) pointed out that ribosomal genes, whose copy number is potentially variable, do not reflect the real pool of organisms in a community and auspicate more rigorous methodologies to assess the ecological questions related to fungal communities. Another limit of DNA metabarcoding is that the number of sequenced species is still a limitation to the precise taxonomic identification of soil fungal sequences.

So far, the meta-genomes studied, included the soil meta-genome, have isolated only bacterial and archaeal genes but not eukaryotic ones (Marmeisse et al., 2017). These authors clearly explain in their review that the reason of this lack is due to the dilution of eukaryotic genes of interest in the total metagenome, the large size of eukaryotic genomes and the strategy of cloning and expressing environmental DNA in bacteria. Besides listing the reasons to care about eukaryotic environmental nucleic acids (most of commercial enzymes and metabolites come from eukaryotes; eukaryotes are highly diverse at both local and global scales and have diverse gene repertoires), Marmeisse et al. (2017) review how metatranscriptomics is an alternative approach to access to environmental eukaryotes, starting with the isolation of their mRNAs that are distinguishable from those of bacteria having the poly-(A) tails lacking in bacteria messages. The systematic sequencing of eukaryotic metatranscriptomes from forest soil has been first applied by Damon et al. (2012), so resulting to be still in its infancy. Although most sequences coming from this approach cannot be affiliated to any taxon, it provides functional data despite of the barcoding of soil communities, in addition to unique products of biotechnological interest. A novel fungal family of oligopeptide transporters has been identified by functional metatranscriptomics of soil eukaryotes by Damon et al. (2011). Within the framework of the DOE Joint Genome Institute Community Sequencing Program, a challenging large-scale metatranscriptomics project - ‘Metatranscriptomics of Forest Soil Ecosystems’^2^ – to explore the interaction of forest trees with communities of soil fungi, including ectomycorrhizal symbionts, has started. This project aims to sequence the metatranscriptome of soil fungi of ecosystems from the boreal, temperate and mediterranean forests. But fungal ecology is also taking benefits from environmental proteomics (Bastida et al., 2009; Schneider et al., 2012; Zampieri et al., 2016). This approach provides insights into the metabolically active species and the composition and functionality of microbial communities. Keiblinger et al. (2016) eloquently review the opportunities and limits of this approach and discuss how linking phylogenetics and functionality can help learn more on microbial ecology and on potential soil metabolic pathways. Within the –omics, also metabolomics has been applied to soil. Jones et al. (2014) obtained the metabolic profiles of communities living in soils from a range of former mine sites in the United Kingdom to assess the effects of pollution. Although each –omics approach provides valuable information separately, only network-based approaches and combination of data can lead to the understanding of microbiomes. Below an example of a combination of data from genomics, metagenomics and metaproteomics.

## Tools From *T. melanosporum* Genome Sequence for Deciphering Its *In Situ* Dynamics

In the last years, the biology and the ecology of truffles have greatly increased, thanks to many new scientific insights and technologies as the sequencing of *T. melanosporum* genome.

Regarding the life cycle, truffles have been considered for long-time self-fertile (Bertault et al., 1998). This opinion could not be tested in absence of an experimental system, based on spore germination, and therefore of the classical breeding of the resulting mycelia (Mello et al., 2005). Only thanks to the *T. melanosporum* genome sequencing, it has been possible to discover that *T. melanosporum* has a heterothallic organization (Martin et al., 2010; Rubini et al., 2011a). Heterothallic organization with a MAT locus structured in two idiomorphs harbored by different strains was also found in other truffles: *T. borchii* and *T. indicum* (Belfiori et al., 2013, 2016). That means that for truffle reproduction it is necessary that strains of opposite mating type meet. Since these discoveries, the spatio-temporal distribution of these strains in soil and in mycorrhizae has been investigated by numerous authors. The distribution of mating type genes of *T. melanosporum* has been first investigated in *T. melanosporum* natural plantation by Rubini et al. (2011b) who reported that, contrary to what is expected, strains with opposite mating types were never present on the same root apparatus, while both mating types were detected in the soil of the plantation. The same authors showed in experiments of inoculation of host plants in controlled conditions that the coexistence of both types on the roots of the same host plant can happen, but lasts until their competition excludes one of the two mating types. According to Iotti et al. (2012) the competition between strains of different mating types seems related to a self-/non-self-recognition system acting before hyphal contact rather than to the presence of a heterokaryon incompatibility (HI) system which leads to the death of the heterokaryotic cells in incompatible reactions. In support of this fact, Rubini et al. (2014) reports that orthologs of the genes controlling HI in other filamentous ascomycetes are present also in the *T. melanosporum* genome, but they lack the key functional domains involved in the HI process. Zampieri et al. (2012) could detect mating type genes for *T. melanosporum* under productive and formally productive trees but, generally, not under unproductive trees, so suggesting that the presence of the two mating types in soil can be a promising predictor of the fertility of truffle orchards and, hopefully of the *T. melanosporum* production when other abiotic and biotic factors are favorable. Mating type distribution of *T. melanosporum* has also been investigated in artificially planted truffiéres in Australia to increase ascoma production (Linde and Selmes, 2012). Since the discovery of the *T. melanosporum* heterothallism, inoculation techniques for production of seedlings with mycelia of opposite mating type are envisaged to improve truffle productivity (Rubini et al., 2014). This modern approach has been recently applied by Iotti et al. (2016) who produced *T. borchii* fruiting bodies starting from the mycorrhization of plants with mycelial pure culture.

**FIGURE 2:**
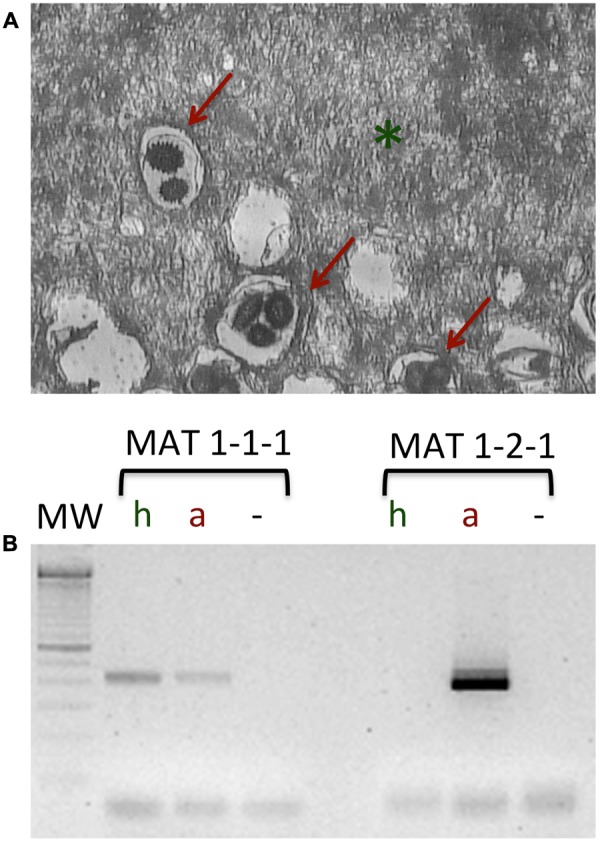
Laser microdissection on the *T. melanosporum* fruiting body as described in Sillo et al. (2013). **(A)** Section of the inner part (gleba) of a *T. melanosporum* fruiting body. The vegetative hyphae (green asterisk) and reproductive structure, i.e., the asci (red arrows) can be distinguished. **(B)** RT-PCR analysis of the microdissected samples (hyphae, h; asci containing the ascospores, a; –, negative control). Using specific primers for MAT1-1-1 and MAT1-2-1, a fragment of the expected size can be observed in both LMD samples (h and a) for the first mating type gene (MAT1-1-1), while the transcripts corresponding to the second mating type gene (MAT 1-2-1) can only be detected in the reproductive compartment (a).

The spatial genetic structure of *T. melanosporum* populations at a small scale has been investigated in two productive *T. melanosporum* orchards, one located in the northern France and the other in central Italy thanks to polymorphic SSR markers searched in the *T. melanosporum* genome and mating type genes (Murat et al., 2013). The analysis of the genetic profiles of ectomycorrhizae using both SSR and mating type markers, and the monitoring of the distribution of *T. melanosporum* mycelia of the two mating types in the soil allowed the authors to demonstrate a pronounced spatial genetic structure of *T. melanosporum*, characterized by non-random distribution of small genets. Several small *T. melanosporum* genets that shared the same mating types could be found on the same host plant, suggesting that the genet distributional pattern is related to the allelic configuration of the MAT locus. However, the factors involved in truffle sexual reproduction are difficult to search due to the impossibility of manipulating truffle *in vitro* (Le Tacon et al., 2015). In *T. melanosporum*, the female gametes are ascogonia (MAT1-1 or MAT1-2) produced by a haploid mycelium forming the ectomycorrhizal root tips from which the peridium and the sterile tissue of the gleba constituting the truffle develop. After the fertilization from germinating ascospores acting as male genotypes, a diploid transitory phase occurs (that cannot be detected in mature ascocarps), followed by a meiosis phase that ends in the formation of a mature truffle (De la Varga et al., 2017). **Figure 2** shows a laser microdissection (LMD) experiment coupled with RT-PCR analysis using mating-type genes on different cell-type populations collected from a *T. melanosporum* fruiting body, i.e., vegetative hyphae and reproductive structures (asci containing the ascospores). As confirmation of the heterotallism in *T. melanosporum* (Rubini et al., 2011a), transcripts corresponding to the first mating type gene (MAT1-1-1) can be observed in both LMD samples, while those corresponding to the second mating type gene (MAT 1-2-1) can only be detected in the reproductive compartment.

From the ecological point of view, the development of the ECM symbiosis and of the fruiting bodies of *T. melanosporum* is associated to the formation of a burnt area (known by the French word brulé), characterized by little vegetation around their host plants because of the phytotoxic effects generated by the truffle metabolites and volatile organic compounds (Splivallo et al., 2011). Metagenomics data applied to French truffle-grounds have showed a reduced fungal biodiversity, a dominance of *T. melanosporum* and a reduced presence both of the ECM Basidiomycota and of bacteria belonging to *Pseudomonas* and Flavobacteriaceae inside the brulé, together with a reduction of richness of arbuscular mycorrhizal fungi (Napoli et al., 2010; Mello et al., 2011, 2013, 2015b; Mello and Zampieri, 2017). In order to relate microbial community composition to ecological processes happening in the brulé, Zampieri et al. (2016) applied a metaproteomics analysis to the brulé previously characterized by metagenomics, and cross-referenced the resulting proteins with a database they constructed, incorporating the metagenomics data for the organisms previously identified in this soil, including the black truffle *T. melanosporum.* The resulting proteins were categorized and assigned to the organisms living in the brulé, leading to discover that the soil inside the brulé contained a larger number of proteins compared with the soil outside the brulé, of which more proteins from herbaceous plants (despite the scarce vegetation typical of such a niche), and more biological processed, mostly of them related to responses to multiple types of stress from most of the brulé components. Thus, although the brulé has a reduced diversity of plant and microbial species, it seems to be a very active environment, characterized by broad stress responses and in particular by herbaceous plants. From these results Zampieri et al. (2016) hypothesize that volatile organic compounds, may elicit stress and defense responses in fungi, bacteria, and above all in the herbaceous plants inside the brulé. At this regard, already Splivallo et al. (2007) had showed that *Arabidopsis*, exposed to volatile organic compounds under laboratory conditions, produced an oxidative burst.

Taking in the all, the combination of metagenomics and metaproteomics has provided a powerful tool to reveal functioning of a complex phenomenon associated to an ECM fungus, as the brulé. Since metaproteomics is the study of all the proteins expressed by the organisms within an ecosystem at a specific time will surely help, together with different -OMIC approaches, to understand the ecological regulation of environmental processes.

## From the Morphological Observations to the Identification of Genes Potentially Involved in the Symbiotic Interface Creation

**FIGURE 3:**
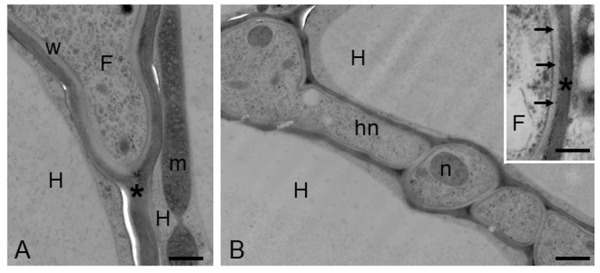
Hazelnut-*T. melanosporum* ectomycorrhizal root. Ectomycorrhizal roots have been prepared for the transmission electron microscopy using the high-pressure freezing and freeze substitution. **(A)** A fungal hypha (F) penetrates between two host cells (H). The asterisk marks the plant cell wall. m, mitochondria; w, fungal cell wall. Bar, 0.60 μm. **(B)** Hartig net (Hn) in a fully developed mycorrhiza. Hyphae develop among plant cells (H), and their cell walls are in direct contact with the plant cell walls, showing a simple interface. Bar, 1.2 μm. (Inset) Magnification of the contact zone between plant (asterisk) and fungal cell wall (arrows). F, fungus. Bar, 0.50 μm.

For many years, the interest of researchers has been dedicated to reveal, through morphological observations, the changes in hyphal growth and remodeling of the root and hyphal cell walls during ECM development (Balestrini and Kottke, 2016). At morphological level, the symbiotic interface in an ectomycorrhiza is formed by the plant and fungal cell walls in direct contact, because the ECM fungus remains apoplastic (Peterson and Massicotte, 2004; Balestrini and Bonfante, 2014; Balestrini and Kottke, 2016; **Figure 3**). The use of *in situ* affinity techniques that utilize specific probes for fungal and plant cell wall components has allowed information to be obtained on the cell wall composition at the plant/fungus interface (Balestrini et al., 1996; Peterson and Massicotte, 2004; Balestrini and Bonfante, 2014; Balestrini and Kottke, 2016). Several fungal proteins localized on the fungal cell wall in the ectomycorrhizal basidiomycete *P. tinctorius* have been observed to be highly increased during eucalypt root colonization, such as symbiosis-regulated acidic polypeptides (SRAPs) and hydrophobins (Laurent et al., 1999; Martin et al., 1999; Tagu et al., 2001). In the ectomycorrhizal ascomycete *T. borchii*, a secreted phospholipase A2 (TbSP1) has been also localized on the fungal cell wall and a role during ECM development has been proposed (Soragni et al., 2001; Miozzi et al., 2005). A homolog gene (*TmelPLA2*) has been also identified in *T. melanosporum* genome and this gene was one of the most upregulated transcripts during the colonization of *Corylus avellana* roots (Balestrini et al., 2012), in agreement with the previous data. More recently, the information derived from the several mycorrhizal genomes sequencing and the transcriptomics data on different ECM symbioses allowed the identification of novel fungal cell wall components with a putative role in the interaction with the host plant. A genome-wide inventory of hydrophobins, i.e., fungal small secreted proteins associated with the outer surface of the cell wall and able to mediate the interaction between the fungus and the environment (Whiteford and Spanu, 2002), have been obtained from *L. bicolor* where it has been demonstrated that the expression of these genes changed depending on the life-cycle stage and on the host root environment (Plett et al., 2012). A weak up-regulation of one of the four putative hydrophobin genes identified in *T. melanosporum* genome, has been also reported in its ECMs (Balestrini et al., 2012). A role for these proteins in the formation of the symbiotic interface and/or in the hyphal aggregation required for the formation of the symbiotic structures has been hypothesized (Balestrini and Bonfante, 2014). Interestingly, genes coding for putative chitin deacetylases (CDAs), which are enzymes belonging to the carbohydrate esterase 4 (CE4) family^3^ that are involved in the chitin conversion to chitosan, have been also reported as upregulated in *T. melanosporum* (Balestrini et al., 2012) and *L. bicolor* (Veneault-Fourrey et al., 2014) ectomycorrhizae, suggesting a role in the symbiosis establishment. The role(s) of CE4 enzymes in ectomycorrhizae is still unknown, but on the basis of their expression profiles, it has been suggested that some of them are involved in cell wall synthesis, whereas others are perhaps involved in fungal colonization to avoid plant defense responses (Veneault-Fourrey et al., 2014). CE4 can be in fact involved in cell wall formation, but a role in the reduction of chitin oligomers elicitor activity through their de-acetylation has been also proposed during plant–pathogen interactions. Additionally, chitin de-acetylation to chitosan in pathogen fungal structures could also have a function to protect fungal cell wall plant from chitinases (Tsigos et al., 2000). The presence of a chitin-binding domain in *TmelPDA3*, one of the *T. melanosporum* upregulated genes, should also be highlighted, considering that a role for chitin-binding proteins has been also proposed in pathogenic fungi to protect the fungal cell wall from chitinases produced by host plants. This has been reported for the biotrophic fungal pathogen *Cladosporium fulvum* that secrets the apoplastic effector Avr4, which is a chitin-binding lectin that functions to protect the integrity of the fungal cell wall against chitinases (van den Burg et al., 2006; Malinovsky et al., 2014). Genome-wide transcriptome profiling allowed to demonstrate that several genes related to cell wall modification, significantly regulated during ectomycorrhiza formation, are involved in fungal cell wall processing, suggesting an extensive remodeling when the fungus is in contact with the host plant cells in agreement with the view of fungal cell wall as a highly dynamic structure (Veneault-Fourrey et al., 2014). Additionally, several expansin-like genes have been identified in the *L. bicolor* genome and several of them were found to be regulated during ECM development, suggesting a role as complement of the enzyme set involved in fungal and/or plant cell wall modification. One expansin-gene (*LbEXP1*) was first showed as the most highly induced CAZyme in ECM tissues (Martin et al., 2008) and then localized on the fungal cell wall, both in the Hartig net and mantle hyphae, suggesting a role in the fungal cell wall remodeling during symbiosis structures development (Veneault-Fourrey et al., 2014).

A subtle remodeling of the root cell wall in response to the contact with the ECM fungus has been reported years ago (Balestrini et al., 1996; Balestrini and Bonfante, 2014 for a review). A localized degradation of pectin has been suggested during fungal colonization, according with the growth of the ECM fungus through the middle lamella and with the expression of fungal genes acting on these plant cell-wall components (Balestrini and Kottke, 2016; Sillo et al., 2016). In addition to a remodeling of the middle lamella, a soft remodeling of the plant cell wall through the loosening of cellulose has been also suggested during *L. bicolor* ECM development. Considering ectomycorrhizae at different stages of the interactions it was possible to verify that *L. bicolor* CAZymes acting on several plant cell wall components are expressed at a different developmental stage (Veneault-Fourrey et al., 2014). Recently, Doré et al. (2017), working on different developmental stages of the ECM interaction between *H. cylindrosporum* and *Pinus pinaster*, showed that genes coding for extracellular proteins, such as MiSSPs and CAZymes, were over-represented among the genes up-regulated upon pre-infectious interaction, suggesting that these specific proteins are host-induced and might play essential function(s) in the early fungal response to host root. Remarkably, the expression of some of these genes has been reported in the Hartig net compartment in *T. melanosporum* and *C. avellana* ECMs (Hacquard et al., 2013). Looking at the plant genes, Sebastiana et al. (2014) also reported that several genes coding enzymes involved in cell wall biosynthesis and modification were found to be differentially expressed in ectomycorrhizal cork oak roots with respect to non-colonized roots. In detail, cell wall-related glycosylhydrolases (GH), which are required for the modification of cell wall polysaccharides and are involved in wall loosening and elongation, were mostly up-regulated in oak ECM roots. By contrast, cell wall-related glycosyltransferases (GT), involved in the synthesis of non-cellulosic polysaccharides as part of the biosynthetic machinery to synthesize the complex plant cell-wall polysaccharides, resulted to be mostly down-regulated. Overall, these plant regulated genes might be involved in the remodeling of the plant cell wall required to facilitate hyphae penetration between cells and fungal accommodation (Sillo et al., 2016), but they might be also involved in maintaining the cell wall thickness during the changes in the architecture of host colonized cells, i.e., radial elongation (Sebastiana et al., 2014), requiring the addition of newly synthesized polysaccharides. Interestingly, an activation of cellulose synthesis in oak colonized roots has been also suggested, and transcripts corresponding to plant expansins, which are known to be involved in cell wall loosening and cell enlargement, were found to be up-regulated in ECM roots (Sebastiana et al., 2014).

However, although many studies suggest a role for the proteins regulated in symbiosis, functional analyses with the aim to highlight the function of these proteins are still lacking, as well as the nature of the cell wall remodeling during the ectomycorrhiza establishment and development.

## Conclusion and Perspectives

In conclusion, genomic and transcriptomic sequencing projects starting with the first mycorrhizal genome sequencing (i.e., that of *L. bicolor*) have allowed the identification of the common core of ECM symbiosis-related genes, as determinants of the symbiotic lifestyle, as well as the identification of species-specific traits. However, genome sequencing is only the first step to obtain information on how an organism interacts with the environment and with other organisms. The combination of functional, structural, cellular, and bioinformatics approaches is providing knowledge on the function of genes/proteins and permits to reconstruct the pathways of an organism in specific growth conditions, and in its natural environment. In fact, metagenomics, metatranscriptomics and metaproteomics studies are currently and fast providing a powerful mean for the analysis of environmental microorganisms without the need of culturing them. At the question: “can -omics provide insight into microbial ecology that cannot be achieved using traditional methods?”, Jansson and Prosser (2013) and Prosser (2015) reply that although -omics generate a large amount of ‘big data’ experiments that test hypotheses on microbe-environment associations may allow more direct identification and analysis of the ecological processes. The large volume of sequence data involved in the ECM symbiosis, provide a reference database for an estimation of the ECM fungal taxa number and their ecology.

The next crucial research will be linking molecular and metabolic data to key processes such as the exchange of nutrients, the plant protection against stresses and diseases and the genes responsible of the symbiosis.

## Author Contributions

RB has contributed on the molecular and cellular interactions in the ectomycorrhizal symbiosis, with particular attention on the cell walls of the two symbionts. AM has contributed on the biodiversity of ectomycorrhizal fungi, their population dynamics and their interactions with other soil microorganisms.

## Conflict of Interest Statement

The authors declare that the research was conducted in the absence of any commercial or financial relationships that could be construed as a potential conflict of interest.

## Footnotes

1 http://scata.mykopat.slu.se/

2 http://mycor.nancy.inra.fr/blogGenomes/?page_id=3262

3 http://www.cazy.org/CE4.html
